# Genetic Screen Reveals the Role of Purine Metabolism in *Staphylococcus aureus* Persistence to Rifampicin

**DOI:** 10.3390/antibiotics4040627

**Published:** 2015-12-07

**Authors:** Rebecca Yee, Peng Cui, Wanliang Shi, Jie Feng, Ying Zhang

**Affiliations:** Department of Molecular Microbiology and Immunology, Bloomberg School of Public Health, Johns Hopkins University, Baltimore, MD 21205, USA; E-Mails: ryee2@jhu.edu (R.Y.); keanuc@163.com (P.C.); wshi3@jhu.edu (W.S.); jfeng16@jhu.edu (J.F.)

**Keywords:** *Staphylococcus aureus*, persisters, purines, rifampicin

## Abstract

Chronic infections with *Staphylococcus aureus* such as septicemia, osteomyelitis, endocarditis, and biofilm infections are difficult to treat because of persisters. Despite many efforts in understanding bacterial persistence, the mechanisms of persister formation in *S. aureus* remain elusive. Here, we performed a genome-wide screen of a transposon mutant library to study the molecular mechanisms involved in persistence of community-acquired *S. aureus*. Screening of the library for mutants defective in persistence or tolerance to rifampicin revealed many genes involved in metabolic pathways that are important for antibiotic persistence. In particular, the identified mutants belonged to metabolic pathways involved in carbohydrate, amino acid, lipid, vitamin and purine biosynthesis. Five mutants played a role in purine biosynthesis and two mutants, *purB*, an adenylosuccinate lyase, and *purM*, a phosphoribosylaminoimidazole synthetase, were selected for further confirmation. Mutants *purB* and *purM* showed defective persistence compared to the parental strain USA300 in multiple stress conditions including various antibiotics, low pH, and heat stress. The defect in persistence was restored by complementation with the wildtype *purB* and *purM* gene in the respective mutants. These findings provide new insights into the mechanisms of persistence in *S. aureus* and provide novel therapeutic targets for developing more effective treatment for persistent infections due to *S. aureus*.

## 1. Introduction

*Staphylococcus aureus* (*S. aureus*) infections cause an intense burden on healthcare throughout the world, as the methicillin-resistant *S. aureus* (MRSA) strain accounts for a majority of the infections present in hospital environments [1]. Infections with MRSA are difficult to treat due to their resistance to multiple antibiotics and highly invasive nature [2]. In particular, USA300 is a MRSA strain that was first isolated in infections among football players in the state of Pennsylvania in the United States in 2000 and has since been discovered in Europe, South America and Australia [3,4]. This invasive strain has been shown to cause infections in community-associated infections, especially in traditionally low risk groups such as children in daycare, inmates in prisons, and military officials [5]. Many *S. aureus* infections, including those caused by USA300, can develop into persistent and recurrent infections such as endocarditis and biofilm infections due to the presence of bacterial persisters [6,7].

Persisters are quiescent organisms that survive exposure to bactericidal drugs and stresses but are still susceptible to drugs and stresses upon exiting dormancy [7]. Persisters are genetically identical to the rest of the population of cells but exhibit a phenotype that allows them to be drug-tolerant [7]. Most studies on persistence mechanisms are done in *Escherichia coli* (*E. coli*). Thus far, pathways such as toxin-antitoxin, SOS response and DNA repair, signal transduction, membrane stress, energy production, phosphate metabolism, and protein degradation are important for persister formation [7].

A majority of the literature that implicates mechanisms of persister formation in *S. aureus* stems from understanding the development of small colony variants (SCVs) that are isolated in persistent infections from patients. The unstable nature of SCVs that revert back to the normal phenotype provides a mechanism for relapsing infections [8]. Earlier works suggest that electron transport and thymidylate biosynthesis were associated with SCV formation [9]. SCVs have defects in genes involved in the respiratory chain such as *hemB*, which is involved in hemin biosynthesis, and *menB*, which is involved in menaquinone production [9]. Additionally, *S. aureus* can form cell-wall deficient bacteria called L-form bacteria, named after the Lister Institute, which is where these morphological variants were discovered, that can evade antibiotic activity and the host immune response [10]. Using a transposon mutant library of *S. aureus* to identify mutants defective in unstable L-form formation, Han *et al.* identified that *glpF* involved in glycerol uptake was critical for L-form formation and persistence to antibiotics in *S. aureus* [11]. Although SCV and L-form bacteria are implicated in causing relapsing and persistent infections, the mechanisms of *S. aureus* persister formation are largely unknown.

Since the discovery of the persister phenomenon in Staphylococcal cultures in 1940s, there is renewed interest in understanding *S. aureus* persistence and persister biology [7]. Here, we performed a systematic, high-throughput mutant screen against mutants of all non-essential genes in the clinically relevant USA300 *S. aureus* strain to identify genes that play a role in bacterial persistence in *S. aureus*. Mutants involved in metabolite production or regulation were found to be required for bacterial persistence to stresses and antibiotics.

## 2. Results

### 2.1. Identification of New Persister Genes

**Figure 1:**
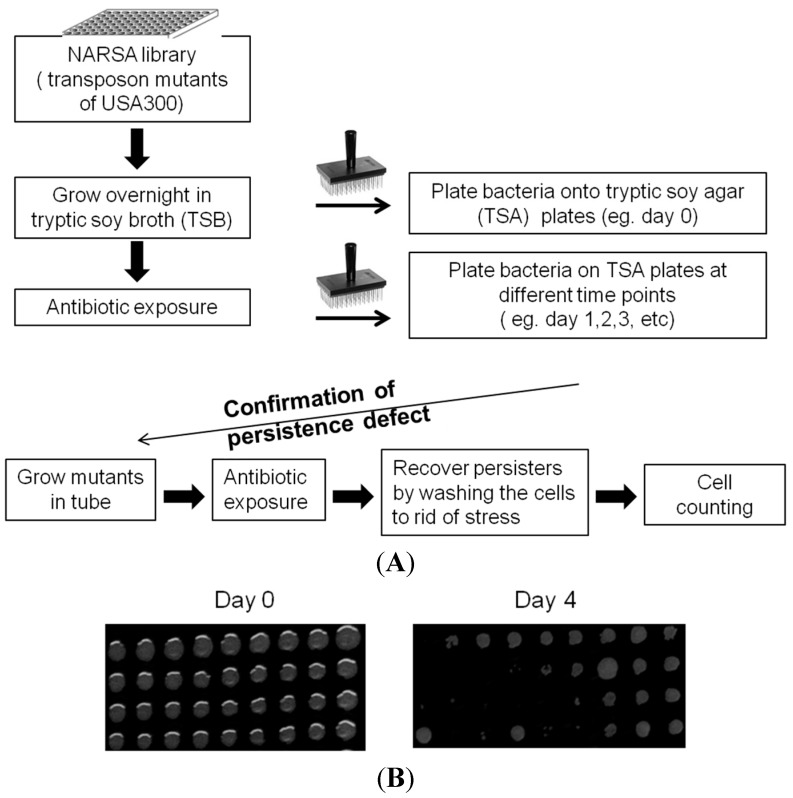
(**A**) Work flow for isolation of mutants with defect in persistence. The *S. aureus* USA300 transposon library from NARSA was grown in tryptic soy broth for 16 h to reach stationary phase. Rifampicin was added to the stationary phase culture at 2 μg/mL (>10× MIC). Before rifampicin exposure, bacteria were stamped onto tryptic soy agar plates (TSA) using a 384-pin replicator as the input control. Over the course of a 4-day antibiotic exposure, bacteria were washed and plated onto TSA plates daily and all mutants that showed defective growth was recorded and tallied until Day 4; (**B**) Representative agar plate images with mutant library after the 4-day rifampicin exposure where some mutants show defect in growth.

To better understand the mechanisms of persister formation, we performed a genetic screen using the Network on Antimicrobial Resistance in *Staphylococcus aureus* (NARSA) mutant library, which contained mutations in all the 1952 non-essential genes in the genome of the USA300 strain [12]. We exposed all mutants in the library to rifampicin, which is a bactericidal agent for *S. aureus* that has been used to treat serious MRSA [13,14]. Persister isolation and analysis is shown schematically in Figure 1. The stationary phase culture was exposed to rifampicin (2 μg/mL, >10× MIC). Using a 384-well pin replicator, bacteria were stamped onto tryptic soy agar (TSA) plates and mutants with defective persistence, as indicated by the lack of growth, were recorded (Figure 1B).

**Figure 2:**
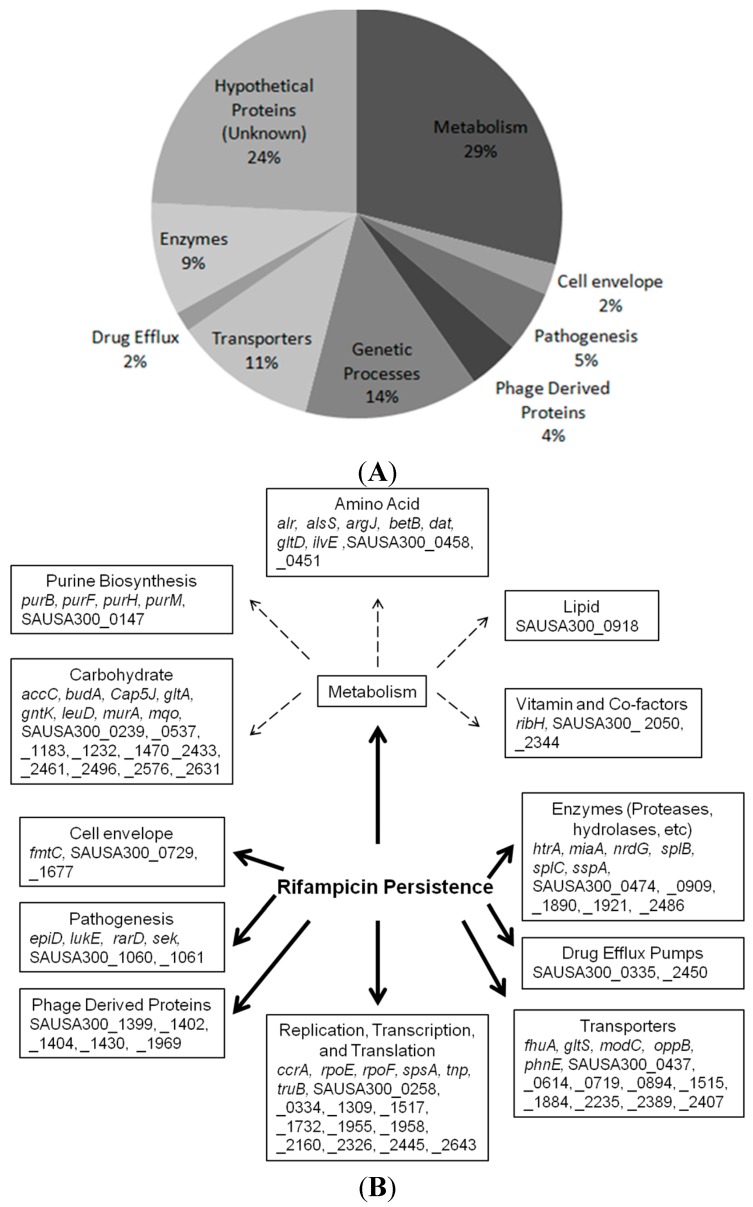

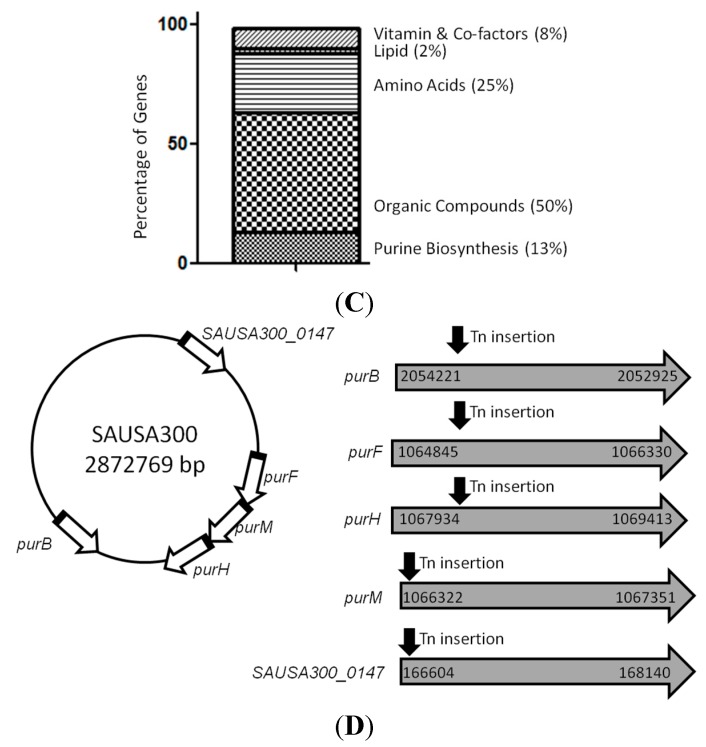
(**A**) Identification and characterization of the 124 genes involved in rifampicin persistence; (**B**) Pathways involved in rifampicin persistence; (**C**) Further breakdown of the metabolic pathways involved in persistence revealed a specific group of mutants belonging to purine biosynthesis; (**D**) The location of the transposon insertion in the genes involved in purine biosynthesis is depicted. All pathway analyses were performed using the Kyoto Encyclopedia of Genes and Genomes (KEGG) database, and the screen results were repeated and were reproducible.

The screen identified 124 mutants that failed to grow on TSA plates after exposure with rifampicin (Figure 2). Of the mutants that showed defective persistence after rifampicin exposure, 29% of them had mutated genes that play a role in the metabolism in pathways such as carbohydrate metabolism, amino acid, and purine biosynthesis. Among the rest of the mutants, 14% of the candidates represented pathways that play a role in genetic processes such as DNA replication and repair, transcription and translation, 11% of the mutants were involved in environmental signaling processes (e.g., transporters and sensor kinases), 9% were enzymes (e.g., proteases, hydrolases, *etc.*), and a minority of the gene hits played a role in bacterial pathogenesis such as toxin production (5%), originated from phage derived proteins (4%), or were involved in cell envelope development and drug efflux pathways (both 2%) (Figure 2A,B).

### 2.2. Mutants Involved in Regulating Purine Biosynthesis are More Susceptible to a Variety of Stresses Including Antibiotics, Heat and Low pH

**Figure 3:**
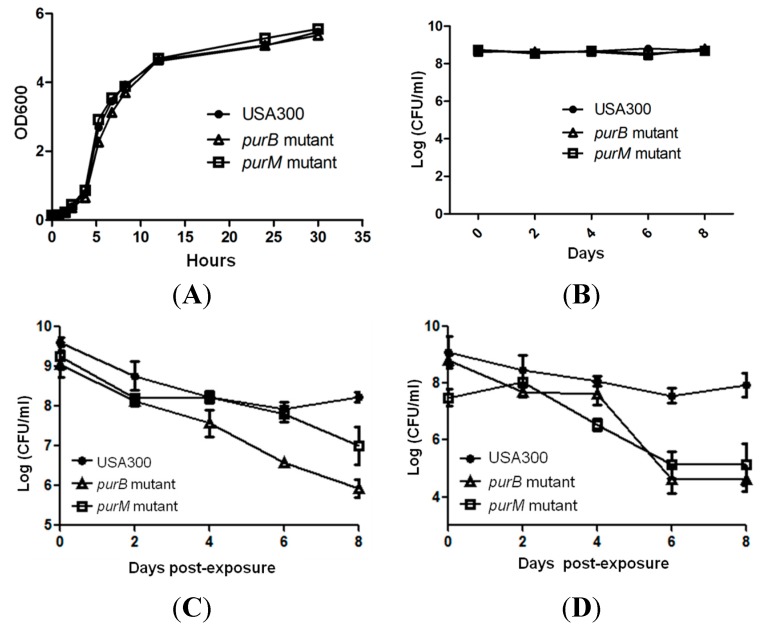

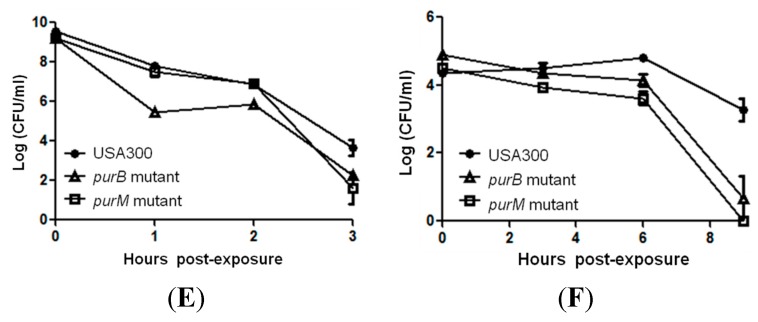
(**A**, **B**) Parental strain USA300 and *pur* mutants showed no defects in log phase or stationary phases under non-stressed conditions. *Pur* mutants had defective persistence in exposure to (**C**) rifampicin (2 μg/mL, >10× MIC), (**D**) gentamicin (60 μg/mL, >10× MIC), (**E**) heat (58 °C), and (**F**) a low pH of 4.0.

Metabolic genes account for 29% of the gene hits that our screen identified. Our data imply that metabolic pathways and genes play a crucial role in persistence. Out of the metabolic genes, a prominent group of mutants, which includes *purB*, *purF*, *purH*, *purM*, and SAUSA300_0147, all play a role in purine biosynthesis (Figure 2C,D). To further validate the role of purine biosynthesis and its possible involvement in persistence and tolerance to antibiotics and stress, we chose two genes, *purB* and *purM*, adenylosuccinate lyase and phosphoribosylaminoimidazole synthetase, respectively, for further confirmation [15]. The *purB* and *purM* mutants not only showed defective persistence to rifampicin (Figure 2) but were also identified as playing a role in persistence to gentamicin using a similarly high throughput method [16]. We further evaluated *purB* and *purM*, since these two genes could potentially be core regulators in persistence. We first performed a growth curve study to exclude the possibility that these mutants have growth defects compared to the parental strain. After we showed that the mutants had no growth defect in log phase and stationary phase under non-stressed conditions (Figure 3A,B), we performed a persister assay, where bacterial cells were first exposed to stresses. Afterward, upon each time point, the stress from the bacteria was removed by several washes and subsequently serial diluted and plated for colony forming unit count (CFU) (Figure 1) [17]. Both of the selected mutants *purB* and *purM* showed increased susceptibility and defective persistence to rifampicin (2 μg/mL, >10× MIC) and gentamicin (60 μg/mL, >10× MIC). Initially, the mutants were killed as much as the parental strain during the first two days but showed increased susceptibility to rifampicin such that, by day 8, the two mutants *purB*, and *purM* had 1 × 10^7^ CFU/mL left, whereas USA300 maintained 1 × 10^9^ CFU/mL (Figure 3C). Under gentamicin (60 μg/mL, >10× MIC) exposure, the remaining CFU/mL of the *purB* and *purM* mutants were 1 × 10^5^ CFU/mL, whereas the parental strain USA300 had 1 × 10^8^ CFU/mL upon 8 days post exposure (Figure 3D). We also determined the minimal inhibitory concentration (MIC) for both rifampicin and gentamicin against the mutants and USA300. Our data show that the MIC was the same for the mutants and USA300, suggesting that neither the mutants nor the parental strain USA300 are resistant to either drug. The MIC for rifampicin and gentamicin is 0.03 μg/mL and 6 μg/mL, respectively. Our study suggests that the mutants compared to USA300 are not any more susceptible to the drugs.

To determine the effect of heat on the survival of the mutants, we subjected the mutants and the parental strain to a heat stress at 58 °C. The mutants were much more sensitive to heat treatment, as demonstrated by the poorer growth seen in the *purB* and *purM* mutants, compared to USA300 (Figure 3E). Compared to all of the other drug and stress exposures, the starting bacterial inoculum size for low pH stress was lowered in order to prevent neutralization of the media by large inocula. Nonetheless, all of the mutants were also more sensitive than USA300 to buffered low pH solutions of 4.0. At a low pH of 4.0, no viable bacteria were recovered from the mutants after 10 h of exposure, whereas the parent strain had about 1 × 10^3^ CFU/mL surviving bacteria (Figure 3F). Throughout the course of both the heat and low pH exposure experiments, the poorer survival in all of the mutants was evident.

### 2.3. Complementation Studies to Confirm the Role of Purine Pathways in Persistence

**Figure 4:**
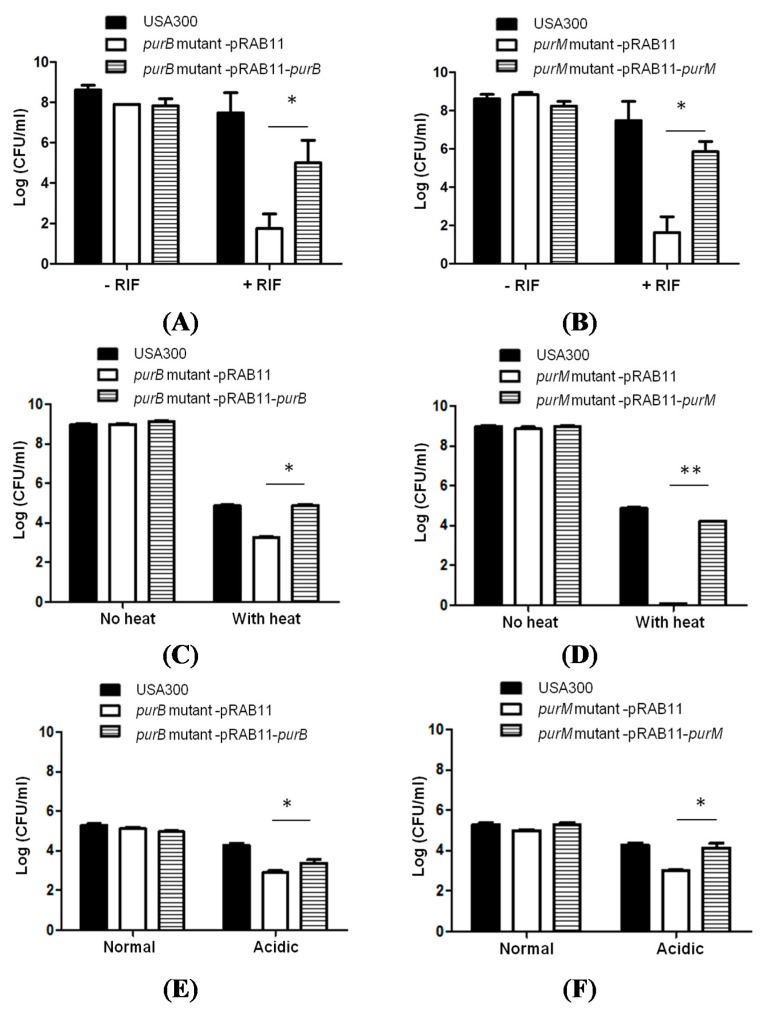
Restoration of persistence phenotype in complemented mutants. Survival of stationary phase cultures of *S. aureus purB* and *purM* mutants transformed with vector pRAB11 or pRAB11-wildtype *purB* and *purM* and the parent strain USA300 upon (**A**, **B**) rifampicin exposure (2 μg/mL, >10× MIC) at 5 days post-exposure; (**C**, **D**) heat exposure (58 °C for 40 min) and (**E**, **F**) low pH of 4.0 for 9 h. The *S. aureus purB* and *purM* mutants complemented by their corresponding wildtype genes partially restored the persistence phenotype of the mutants (Student *t*-test, * *p*-value < 0.05, ** *p*-value < 0.005). No change in persistence was observed in stress-free conditions over the course of 5 days.

To confirm that *purB* and *purM* are responsible for defective persistence, we complemented the *purB* and *purM* mutants with the wildtype *purB* and *purM* gene, respectively, using the *S. aureus-E.coli* shuttle vector pRAB11 (Table 1). The shuttle vector pRAB11 harbors a *tet* operator that is induced by anhydrotetracycline (ATc) [18]. Our findings suggest that both *purB* and *purM* are important for rifampicin persistence (Figure 4). Under prolonged rifampicin exposure, the complemented *purB* mutant restored partial persistence to rifampicin on day 5 (Figure 4A) with an average of 1 × 10^4^ CFU/mL compared to the parental strain that harbored 1 × 10^7^ CFU/mL. The *purB* mutant transformed with the pRAB11 vector control showed a defect in persistence, and no difference was observed among any of the strains under non-stressed conditions (Figure 4A). Similarly, the complemented *purM* mutant restored rifampicin persistence at a level similar to the parental strain. On day 5, the complemented *purM* mutant had an average of 1 × 10^6^ CFU/mL compared to the parental strain that had 1 × 10^7^ CFU/mL (Figure 4B). The *purM* mutant transformed with the pRAB11 vector control had no colonies by day 5, and no phenotypic difference appeared under non-stressed conditions (Figure 4B). The complemented *purB* mutant and *purM* mutant both restored persistence in heat exposure (58 °C) (Figure 4C,D). After forty minutes of heat exposure, the *purB-*complemented mutant and *purM*-complemented mutant had roughly 7.8 × 10^4^ CFU/mL and 1.64 × 10^4^ CFU/mL, respectively, which is comparable to USA300, which had 7.5 × 10^4^ CFU/mL. Additionally, the complemented *purB* mutant and *purM* mutant restored persistence when exposed to a low pH of 4 (Figure 4E,F). After 9 h of exposure to a pH of 4.0, the *purB* complemented and *purM* complemented mutants had roughly 0.4 × 10^4^ CFU/mL and 2.5 × 10^4^ CFU/mL, respectively, comparable to USA300, which had 2.1 × 10^4^ CFU/mL. In contrast, the mutants harboring the empty vector control had a significantly lower number of cells under heat and acid stress conditions compared to the respective complemented strains.

**Table 1:** Primers used in this study. The underlined sequences AGATCT and GAATTC represent the BglII and EcoRI restriction sites incorporated for cloning the wildtype gene into shuttle vector pRAB11 for complementation.

Primer Name	Sequence	Source or Reference
purBF	5′-GCAAGATCTATGATTGAACGCTATTCTAG-3′	This study
purBR	5′-ACGGAATTCTTATGCTAATCCAGCGCGTTCG-3′	This study
purMF	5′-GCTAGATCTATGTCTAAAGCATATGAACAATC-3′	This study
purMR	5′-ACGGAATTCTTATACCCCCAACAATTCAAT-3′	This study

## 3. Discussion

Despite the discovery of *S. aureus* persisters in 1944 and the prevalence of persistent infections caused by *S. aureus*, the molecular mechanisms underlying *S. aureus* persister survival has remained largely unknown [19]. The convenience of a comprehensive transposon-mutant library has allowed us to perform the first whole genome-wide mutant analysis and to provide insight into the molecular basis of *S. aureus* persisters. This study represents the first systemic analysis of persister genes and pathways in a circulating clinical isolate of *S. aureus*.

We identified 124 different mutants that showed defects in rifampicin persistence. A majority of the mutants belongs to metabolite production and regulation pathways such as purine biosynthesis. Our findings revealed that five purine biosynthesis genes (*purB*, *purF*, *purH*, *purM*, and SAUSA300_0147) are involved in *S. aureus* persistence. In particular, we chose *purB* and *purM*, which encode adenylosuccinate lyase and phosphoribosylaminoimidazole synthetase respectively, for confirmation, and, indeed, complementation studies show that genes involved in purine biosynthesis are important for antibiotic and stress tolerance in *S. aureus*. Although the molecular mechanisms by which purine pathway mediate persistence remain to be determined, our findings are consistent with previous research suggesting that defective purine synthesis has decreased biofilm formation and attenuated virulence in persistent infections such as endocarditis [20]. In addition, purine biosynthesis mutants (*purL* and *purM*) of *Burkholderia* fail to colonize in the host symbiotic organ and exhibit decreased biofilm formation [21]. The role of *pur* genes in Staphylococcal persistence is novel, and future animal studies are needed to confirm the virulence and persistence of the *S. aureus pur* mutants *in vivo*.

**Figure 5:**
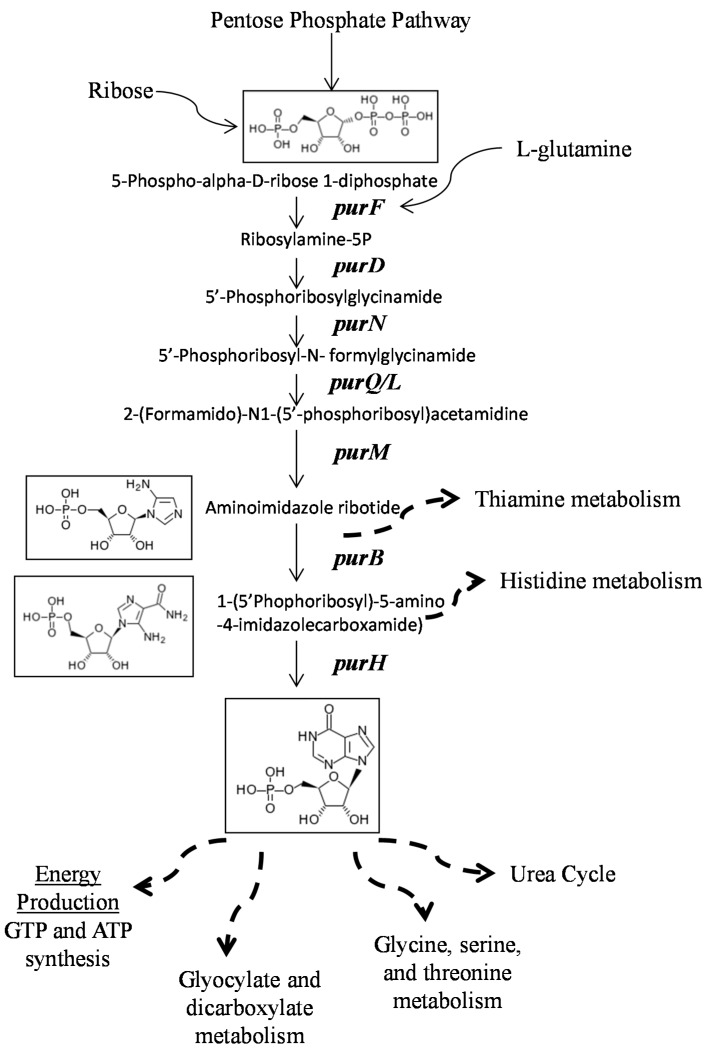
The schematic diagram of the *pur* pathway. The disruption of genes (*purB*, *F*, *H*, *M*) involved in the purine biosynthesis pathway may cause defects in downstream energy production, amino acid biosynthesis and urea cycle, which may be responsible for the defect in persistence observed in this study to rifampicin and other antibiotics and stresses.

Several mechanisms may be underlying the defect in persistence seen in purine biosynthesis mutants. For example, a defect in the purine biosynthesis pathway may lead to decreased downstream energy production, amino acid biosynthesis and urea cycle activation, which may be responsible for the defect in persistence observed in this study to rifampicin and other antibiotics and stresses (Figure 5). Purine biosynthesis has also been shown to be associated with survival in stressed conditions such as vancomycin and daptomycin in other strains of *S. aureus* [22,23]. The final step in purine nucleotide synthesis leads to AMP formation, increased AMP, and thus ATP energy levels, which were observed in antibiotic resistant strains [23]. It is hypothesized that increased purine biosynthesis would allow for more energy used in generating polymers, one of the most energy demanding process in bacteria. In *S. aureus* and other Gram-positive bacteria, the peptidoglycan layer is the most abundantly large polymer [23]. While mechanisms regarding purine biosynthesis in persistence remain to be further elucidated, our hypothesis is consistent with the participation of other genes in persistence from our data. Mutants such as *accC*, *budA*, *Cap5J*, *gltA*, *gntK*, *leuD*, *murA*, *mqo*, each of which play a role in carbohydrate metabolism, SAUSA300_0918, which plays a role in lipid metabolism, *fmtC*, SAUSA300_0729, and SAUSA300_1677 in cell wall and membrane synthesis all showed defects in rifampicin persistence, which suggests a possible role of purine metabolism and of cell envelope formation in persister formation.

Other general mechanisms involved in persistence may help explain the role of purines in persister biology. Studies have shown that pathways involved in DNA repair mechanisms, SOS response, and energy production affect persister survival [7,24]. Purines are important substrates for both DNA synthesis and hence will alter DNA repair processes that make it difficult for the bacteria to repair their genetic material upon stress damage. Additionally, purines are starting compounds for GTP synthesis and thus play an important role for energy production in regulating cell growth [25]. Bacterial cells can be sent into a dormant-like stage through modulation of energy and alarmone (p)ppGpp production [26]. However, imbalance GTP synthesis and metabolism is associated with decreased (p)ppGpp synthesis. Since purines play a crucial role in producing (p)ppGpp substrates, our *pur* mutants may experience the lack of (p)ppGpp modulation into a persistence state. This phenomenon has been shown in *Pseudomonas*, where (p)ppGpp levels affect the persistence in starvation, biofilm formation, and oxidative stress [27]. Similar mechanisms may explain why different *pur* mutants were unable to persist in our screen.

While our studies focused primarily on genes involved in purine biosynthesis, it is important to note that, based on our screen, metabolic processes showed the largest role in rifampicin tolerance and should be further explored in future studies. For example, arginine biosynthesis has recently been proposed as contributing to successful *S. aureus* infection [28]. Most research focused on genes encoded by the arginine catabolic mobile element (ACME), which does not include *argJ*, an acetyltransferase [15,29] that was identified as important to persistence based on our screen. Nonetheless, studies suggested that arginine synthesis might be pivotal in allowing *S. aureus* to colonize the skin and survive in abscesses [28]. The bacterial cells use arginine to increase the pH by ammonia production of the extracellular milieu on the skin, which may aid the survival of *S. aureus* [28,29]. As another example, *gltS*, which encodes a sodium/glutamate symporter, has been shown to be upregulated during biofilm formation [30]. Glutamate is required for the development of arginine and produce byproducts such as ornithine. Ornithine is a pivotal compound in cell metabolism in making prolines, polyamines, antibiotics, proteins, and peptidoglycan. The products driven by ornithine synthesis enhance bacterial growth and pathogenesis [31]. As for the *gltS* transporter, acquisition of amino acid glutamate allows for the production of NH_3_ in the periplasm of the cell to allow for the bacteria to adapt to the acidic environment, similar to the skin [32]. The role of glutamate in regulating bacterial stress includes both the upregulation of *GcrR* [33], which controls the acid tolerance resistance in *Streptococcus mutans*, and the downregulation of *MarR*, which regulates virulence factors, in response to antibiotics and oxidative stresses [34].

Despite the significant findings of this study, there are some limitations. First, due to *S. aureus*’s ability to cause wound infections, endocarditis and systemic disease [6,7], different animal models (e.g., skin model, intraperitoneal injection, and murine model for urinary tract) should be considered to validate our findings [35,36,37]. These will be addressed in future studies. Secondly, a mutant screen only explores persister formation at the DNA and RNA level. Further proteomic, metabolomic and even epigenetic analyses (as our screen indicated genes with acetyl-, and methyl-transferase activities to be important) would offer more comprehensive insight into the effects of gene mutations on persistence. Thirdly, while this screen is comprehensive in the sense that all the non-essential genes of the clinical isolate of MRSA are included, their effects on the persistence of these mutants are determined by the conditions of our assays. Persistence can also be affected by variables such as different aged cultures, concentrations of drugs and bacteria, inoculum size, and the length of the drug exposure [17]. Our screen reveals potential candidates specific to our conditions and may underscore certain genes that can still play a role despite not being revealed from our screen. We performed our complementation studies using an *E. coli-S. aureus* shuttle vector pRAB11, which contains an anhydrotetracycline (ATc) inducible promoter [18]. The reason to use a vector with an inducible promoter is to rule out possible unwanted regulation by the bacterial host and to examine the effect of the inducible gene on persister formation more clearly and effectively. We acknowledge that using an inducer Atc introduces a variable that could complicate data analysis. Future studies will include the use of the constructs with native promoters. We included a negative control by introducing the empty vector into a mutant, and our data suggests that there is a statistically significant difference (using student *t*-test) in persistence between the empty vector control and the complemented mutants. Lastly, secondary mutations may have occurred in some mutants that could affect the phenotypic outcomes. However, our results on the mutant phenotypes are reproducible, confirmed by complementation studies, and the same subculture from the same stock was also used each time to ensure reproducibility among all replicates.

## 4. Experimental Section

### 4.1. Culture Media, Antibiotics, and Chemicals

Ampicillin, chloramphenicol, rifampicin, gentamicin, and erythromycin were obtained from Sigma-Aldrich Co. (St. Louis, MO, USA). Stock solutions were prepared in the laboratory, filter-sterilized and used at indicated concentrations. Bacterial strains and plasmids used in this study include the library of transposon mutants of USA300, a clinical MRSA isolate, offered by the Network on Antimicrobial Resistance in *Staphylococcus aureus* (NARSA). *S. aureus* strains were cultivated in tryptic soy broth (TSB) and tryptic soy agar (TSA) with the appropriate antibiotics and growth conditions, as mentioned, and *E. coli* strains were cultivated in Luria-Bertani (LB) broth or agar at 37 °C.

### 4.2. Library Screens to Identify Mutants with Defect in Persistence

The NARSA library consisting of 1952 transposon mutants of *S. aureus* USA300 was grown in TSB containing 50 μg/mL erythromycin at 37 °C in 384-well plates overnight without shaking. The library was grown to stationary phase in tryptic soy broth (TSB) with erythromycin, the antibiotic selective marker of the mutants from the NARSA library [12]. Rifampicin at 2 μg/mL was added to overnight cultures in the wells. The plates were further incubated for 24 h when the library was replica transferred to TSA plates to score for mutants that failed to grow after drug exposure. The antibiotic exposure was carried out over of a period of at least 6 days.

### 4.3. Susceptibility of Mutants to Various Antibiotics and Stresses

The susceptibilities of stationary-phase mutants and the parent strain USA300 cultures to antibiotic rifampicin (2 μg/mL, >10× MIC) and gentamicin (60 μg/mL, >10× MIC) were evaluated in drug exposure experiments. The antibiotic exposure was carried out over the course of 8 days at 37 °C without shaking. The susceptibilities of the mutants and parent strains to low pH stress was tested by diluting overnight culture 1:100 in citric acid buffer, pH 4, at 37 °C. To test susceptibilities to heat, undiluted overnight cultures were placed into a bathtub of water at 58 °C. At different time points, aliquots of bacterial cultures exposed to the antibiotics were taken out, washed in saline, serial-diluted, and plated for viable bacteria (cell forming unit, CFU) on TSA plates.

### 4.4. Complementation of S. aureus Mutants

The wildtype genes of interest, *purB*, and *purM* from *S. aureus* USA300, were amplified by PCR using primers listed in Table 1. The PCR primers contained restriction sites BglII and EcoRI. The PCR parameters were: 94 °C for 15 min, followed by 35 cycles of 94 °C for 30 s, 55 °C for 30 s, and 72 °C for 2 min, followed by a final extension at 72 °C for 10 min. The PCR products were digested with BglII and EcoRI and were cloned into plasmid shuttle vector pRAB11, which harbors a *tet* operator that is induced by anhydrotetracycline (ATc) [18], and were cut with the same enzymes. The ligated products were chemically transformed into DH5alpha cells and spread on LB plates containing 100 μg/mL ampicillin and grown overnight at 37 °C. Colonies were selected for the correct construct, isolated for introduction into *S. aureus* RN4220 by electroporation (voltage = 2.5 kV, resistance = 100 Ω, capacity = 25 μF) using a MicroPulser Electroporation Apparatus (Bio-Rad, Hercules, CA, USA), spread onto TSA plates containing 10 μg/mL chloramphenicol, and grown overnight at 37 °C. Afterward, pRAB11 plasmid DNA was isolated from RN4220, and along with the pRAB11 vector alone control was then introduced into the respective *S. aureus* USA300 mutant strain by electroporation. Positive clones were identified by restriction digestion, PCR, and DNA sequencing.

For complementation of the *purB and purM* mutants, *purB* mutant-pRAB11 vector control, *purB* mutant pRAB11-*purB* complemented strain, *purM* mutant-pRAB11 vector control and the *purM* mutant pRAB11-*purM* complemented strain were grown in TSB broth with 5 μg/mL chloramphenicol overnight to stationary phase. The strains were then refreshed into 1:100 TSB only and grown to log phase at OD_600_ of 0.6. The cells were subsequently induced with 25 ng/mL of anhydrotetracycline. Cells were then washed twice with PBS and then re-suspended into MOPS buffer to perform drug exposure and persister assays as described [17].

## 5. Conclusions

In conclusion, we report the molecular basis of persistence in *S. aureus*. Our study encompasses the consideration of all the non-essential genes in *S. aureus*, and, to our knowledge, this is the first report on identification of *S. aureus* persistence genes from a whole genome perspective. Our high throughput protocol identified many mutants with defects in persister formation and survival, and the selection of several mutants validated our findings. Persister bacteria pose enormous public health problems due to relapse and can cause the development of genetic drug resistance from prolonged treatment with antibiotics. Our studies will not only help understand new mechanisms underlying persister formation but also provide novel therapeutic and vaccine targets for developing more effective treatment and prevention for *S. aureus* infection.
